# Parent-implemented augmented communication intervention and young children with Down syndrome: an exploratory report

**DOI:** 10.3389/fpsyg.2023.1168599

**Published:** 2023-06-13

**Authors:** MaryAnn Romski, Rose A. Sevcik, Andrea Barton-Hulsey, Evelyn Fisher, Marika King, Phebe Albert, Gal Kaldes, Casy Walters

**Affiliations:** ^1^Departments of Communication and Psychology, Georgia State University, Atlanta, GA, United States; ^2^Department of Psychology, Georgia State University, Atlanta, GA, United States; ^3^Department of Communication Sciences and Disorders, Florida State University, Tallahassee, FL, United States; ^4^Center for Development and Disability, University of New Mexico, Albuquerque, NM, United States

**Keywords:** Down syndrome, language development, speech development, augmentative and alternative communication (AAC), early intervention

## Abstract

**Introduction:**

Young children with Down syndrome (DS) present with speech and language impairments very early in childhood. Historically, early language intervention for children with DS included manual signs, though recently there has been an interest in the use of speech-generating devices (SGDs). This paper examines the language and communication performance of young children with DS who participated in parent-implemented communication interventions that included SGDs. Specifically, we compared the functional vocabulary usage and communication interaction skills of children with DS who received augmented communication interventions (AC) that included an SGD with those children with DS who received spoken communication intervention (SC).

**Methods:**

Twenty-nine children with DS participated in this secondary data analysis. These children were part of one of two longitudinal RCT studies investigating the effectiveness of parent-implemented augmented communication interventions in a larger sample of 109 children with severe communication and language impairments.

**Results:**

There were significant differences between children with DS in the AC and SC groups in terms of the number and proportion of functional vocabulary targets used and the total vocabulary targets provided during the intervention at sessions 18 (lab)and 24 (home).

**Discussion:**

Overall, the AC interventions provided the children with a way to communicate via an SGD with visual-graphic symbols and speech output, while the children in the SC intervention were focused on producing spoken words. The AC interventions did not hinder the children’s spoken vocabulary development. Augmented communication intervention can facilitate the communication abilities of young children with DS as they are emerging spoken communicators.

## Introduction

Children with Down syndrome (DS) are known to present with speech and language impairments (Abbeduto et al., 2007; Romski et al., 2021; Wilkinson and Feinstack, 2021). These speech and language impairments are evident very early in childhood with the delayed onset of babbling followed by a gap between receptive and expressive language development (Miller, 1999; Fidler, 2005; Romski et al., 2021; Wilkinson and Feinstack, 2021). The most striking finding is that children with DS often have relatively strong receptive language skills but have more significant delays in expressive language skills (Miller, 1999; Warren et al., 2020).

One intervention approach that has been part of early language interventions for young children with DS is the use of augmentative and alternative communication (AAC; Wilkinson and Feinstack, 2021). The American Speech Language Hearing Association (ASHA, 2019) defined AAC as an area of clinical practice that addresses the needs of individuals with significant and complex communication disorders characterized by impairments in speech-language production and/or comprehension, including spoken and written modes of communication. AAC includes unaided and aided forms of communication. Unaided forms of AAC include simple gestures, facial expressions, and other manual signs. Aided forms of AAC include picture communication boards, dedicated computers that talk using a synthetic or digitized voice (also described as speech-generating devices or SGDs), as well as iPads or other tablets with various software applications (Beukelman and Light, 2020).

Historically, the use of manual signs, or unaided AAC, was employed with young children with DS who demonstrated emerging spoken language skills (Bird et al., 2000). Manual signs were thought to serve as a bridge to early receptive and expressive spoken language (Iverson et al, 2008). Studies supported this rationale and found that young children with DS could learn to use manual signs to communicate (Romski and Ruder, 1984; Kouri, 1989; Launonen et al., 1996; Foreman and Crews, 1998; Wright et al., 2013; Kaiser and Hampton, 2017). Key word signing has been used successfully with older children with DS (Frizelle and Lyons, 2022). Parents, however, reported a range of issues that may impact the success of key word signing (Glacken et al., 2019). There are two important clinical issues related to manual sign instruction (Romski et al., 2021). First, children with DS have difficulty continually producing intelligible signs given their motor dexterity difficulties. Second, communicative partners of children with DS must learn to understand and produce manual signs, thus potentially limiting the number of communication partners who can understand and use manual signs with this population.

Recent technological advances in aided forms of communication may provide a choice that complements unaided forms of AAC. These aided forms of communication place different motoric demands on the child with DS by having the child point to or touch a symbol on a board or device rather than physically produce a manual sign. When speech output is available, the partners also hear the spoken word, albeit synthetic or digital. In a case study, Iacono and Duncum (1995) found that the use of speech output AAC technology paired with manual signs was more effective than manual signs alone for eliciting single-word productions as well as two- and three-word combinations. In recent years, SGDs have been increasingly used with children with developmental disabilities to support communication development during early intervention and preschool (Romski et al., 2015; Barton-Hulsey et al., 2021). The outcomes of this work suggest positive gains in receptive and expressive communication for children with developmental disabilities. Less attention has been focused specifically on children with Down syndrome and the outcomes associated with the use of SGDs compared to other communication intervention approaches (Barbosa et al., 2018). There are a few studies that focus on the use of SGDs with children with developmental disabilities (Van der Meer et al., 2012; Barbosa et al., 2018). In two randomized controlled trials, Romski et al. (2010, 2023) found that augmented communication interventions that included an SGD and parent coaching had a positive effect on communication for young children with developmental delays who began intervention with less than 10 spoken words. The positive effects included increases in vocabulary size, the spontaneous use of targeted symbol vocabulary, and communication interaction skills. Importantly the augmented communication interventions did not hamper spoken vocabulary development.

While some of these studies found that SGDs are viable for use with children with developmental disabilities, they did not focus exclusively on use with children with DS in the service of language intervention. Additional research is needed to examine the role SGDs can play in early language interventions for young children with DS.

This paper explores the language and communication performance of young children with DS who participated in a larger randomized controlled study of parent-implemented communication interventions that included SGDs. Specifically, compared the functional vocabulary usage and communication interaction skills of children with DS who received augmented communication intervention with those who received spoken communication intervention alone. This study also compared parent communication interaction skills between intervention groups after participating in coaching in the lab and at home.

## Method

### Participants

Twenty-nine children diagnosed with DS were included in a secondary data analysis. These children already participated in one of two longitudinal randomized controlled studies of parent-implemented communication interventions as part of a larger sample of 109 children with severe developmental delays and communication and language impairment that did not separately report on the performance of the 27% of children with DS. Children were recruited from a variety of professional sources in the metropolitan Atlanta area who provided services for children with communication and language impairment, including clinical psychologists, developmental pediatricians, pediatric neurologists, and SLPs. The subset of children with DS included 20 males and 9 females, ages 24 to 29 months (*M* = 28.67, SD = 3.65) at the onset of the study. All children had an expressive language age-equivalent score on the *Mullen Scales of Early Learning* (MSEL; Mullen, 1995) of less than 12 months and a vocabulary of no more than 10 spoken words according to parent reports and clinical observations. Other inclusion criteria were a primary language of English and the presence of sufficient gross motor skills to manipulate an SGD.

A set of developmental assessments, including measures of communication, adaptive behavior, and motor, and visual–spatial reception skills was administered to each child during pre-intervention by a certified SLP who was masked to the child’s group assignment. The baseline developmental assessment provided a description of the children’s developmental and language skills at the onset of the study. Pre-intervention assessment scores for all children with DS were reported by augmented or spoken communication intervention groups in Table 1. Standard scores on the MSEL visual reception and receptive language domains were significantly higher for children in the augmented communication group than the spoken communication group, *p* = 0.012 and *p* = 0.003, respectively. There were no significant differences in expressive communication skills and no other significant group differences on any other baseline measures.

**Table 1:** Pre-intervention assessment scores for children by intervention group.

	Intervention group	
Assessment variable	SC (*n* = 6)	AC (*n* = 23)
Age (months)	28.17 (1.72)	28.78 (4.02)
VABS ABC (SS)	64.17 (6.15)	70.30 (6.47)
MSEL ELC (SS)	51.67 (4.76)	57.78 (9.97)
MSEL visual reception (SS)	21.83 (3.0)	28.74 (10.78)
MSEL fine motor (SS)	24.0 (6.48)	22.74 (5.88)
MSEL receptive language (SS)	21.0 (2.53)	28.13 (9.33)
MSEL expressive language (SS)	22.33 (3.83)	23.70 (5.35)
SICD receptive language (months)	16.67 (3.93)	19.18 (4.73)
SICD expressive language (months)	15.67 (2.66)	18.82 (5.68)
MCDI spoken words	11.83 (10.61)	15.70 (17.70)
MCDI words understood	178.17 (145.05)	132.87 (70.13)

SS, standard score; SC, spoken communication group; months, age equivalent score in months; AC, augmented communication group; VABS, Vineland adaptive behavior scales; MSEL, Mullen scales of early learning; SICD, sequenced inventory of communication development; MCDI, MacArthur-Bates communicative development inventories; Scores are means (with standard deviations in parentheses).

At the time of participation in each of the original studies, 28 of the 29 children with DS (97%) were receiving speech-language therapy for an average of 1.1 (SD = 0.6) hours per week, and 17 children with DS (59%) were reported to be using at least a few manual signs to communicate. It is important to note that the intervention provided in these two studies was supplemental to the clinical services that were received outside of participation in the study.

### Overview of randomized-controlled studies

In the first study, 62 parent–child pairs, including 18 children with DS (29% of the sample), were randomly assigned to a spoken communication intervention or one of two augmented communication interventions that employed the use of an SGD (Romski et al., 2010). In the second study, 47 parent–child pairs, including 11 children with DS (23% of the sample), were randomly assigned to one of two augmented communication interventions (Romski et al., 2023).

#### Description of interventions

The overarching goal of all the interventions was to increase the spontaneous use of the target vocabulary words and to engender improved support for communication interaction between the child and parent at home. In both of the two original studies, parent–child pairs were assigned, via a randomized stratification strategy for etiology and MSEL composite score, to one of four intervention groups: Spoken Communication (SC, DS *n* = 6), Augmented Communication- Input (AC-I, DS *n* = 6), Augmented Communication- Output (AC-O, DS *n* = 12), and Augmented Communication-Input Output Hybrid (AC-IO; DS *n* = 5). As seen in Table 2, all four interventions shared a common structure and vocabulary and the three augmented interventions shared the same mode (using an SGD to include visual-graphic symbols with speech output). The SC intervention focused on developing spoken language vocabulary words. In order to maximize the sample size in the current study, the three augmented communication groups were combined and referred to as the Augmented Communication (AC) group.

**Table 2:** Description of intervention by group.

	Intervention group			
Intervention component	SC	AC-I	AC-O	AC-I/O
Target vocabulary	I/P and child use speech to communicate	I/P uses the SGD to provide comm. Input to child	Child uses the SGD to communicate	I/P uses the SGD to provide comm. Input; the child uses SGD to communicate
Mode	Individualized vocabulary of spoken words	Individualized vocabulary of visual-graphic symbols + words	Individualized vocabulary of visual-graphic symbols + words	Individualized vocabulary of visual-graphic symbols + words
Strategies	I/P encourages and prompts the child to produce spoken words	I/P provides vocabulary models to child using the SGD; Symbols are positioned in the environment to mark referents	I/P encourages and prompts the child to produce communication using the SGD	I/P provides vocabulary models to child by using the device; Symbols are positioned in the environment to mark referents; I/P encourages and prompts the child to produce communication using the SGD
Parent coaching	I provides resource and coaching for P	I provides resource and coaching for P	I provides resource and coaching for P	I provides resource and coaching for P

SC, spoken communication; AC-I, augmented communication-input; AC-O, augmented communication-output; AC-I/O, augmented communication-input/ output; I, interventionist; P, parent. Adapted from Sevcik et al. (2021).

Each intervention was designed to be completed over the course of 12 weeks (two sessions per week), with the first 18 sessions (9 weeks) occurring in the Toddler Language Intervention Project Lab at Georgia State University, and the final 6 sessions (3 weeks) occurring in the children’s homes. Each 30-min intervention session consisted of natural communication interactions during three 10-min activities. The three child-oriented activities were (1) playing with toys, (2) reading/looking at picture books, and (3) eating a snack in that order. These three activities were designed to simulate routines that the parent and child engaged in at home.

As detailed in Romski et al. (2010, 2023), at the beginning of each of the 12 weeks parents received training materials that detailed the parent and child goals for that week. Parents were gradually guided through the activities by the SLP as they observed the session conducted by one of nine trained interventionists (with a bachelor’s degree or higher in communication or psychology) through a one-way viewing window. As the parent backed into the session’s activities beginning with the snack, the interventionist coached them to conduct the activities until they were leading the entire intervention session. See Table 3 for an overview of parental participation across all sessions. The guidance began with parent observation and gradually brought the parents into the intervention sessions. Beginning with parent observation was based on parent feedback during a pilot intervention implementation. As part of using the SGD or spoken intervention strategy, the interventionist and SLP coached the parents to integrate naturalistic communication intervention strategies during the three activities. These strategies created communication opportunities for the child to use target vocabulary during the activities (e.g., offering choices, pause time, and environmental arrangement of toys, books, and snacks to create communication temptations). Parents received guidance and coaching when needed from the interventionist and the SLP throughout the course of the intervention sessions. At the end of each session, the parent, interventionist, and SLP discussed the outcome of the session.

**Table 3:** Sequence of intervention sessions and parent role.

Week/sessions	SLP role	Parent role in session		
		Play	Books	Snack
1/1,2	LAB – Parent observed the interventionist communicating with the child during the three activities as the SLP described the intervention to the parent and answered parent questions	O	O	O
2/3,4	LAB – Parent and SLP observed session run by interventionist	O	O	O
3/5,6	LAB – Parent and SLP observed session run by interventionist	O	O	O
4/7,8	LAB – Parent and SLP observed session run by interventionist	O	O	O
5/9,10	LAB – Parent and SLP observed session run by interventionist and parent joined the session for snack	O	O	X
6/11,12	LAB – Parent and SLP observed the session run by the interventionist and the parent joined the session for book + snack	O	X	X
7/13,14	LAB – Parent joined the entire session with the interventionist	X	X	X
8/15,16	LAB – Parent led the entire session; intervention supported parent as needed	X	X	X
9/17,18	LAB – Parent led the entire session; intervention supported parent as needed	X	X	X
10/19,20	HOME – Parent led the entire session; intervention supported parent as needed	X	X	X
11/21,22	HOME – Parent led the entire session; intervention supported parent as needed	X	X	X
12/23,24	HOME – Parent led the entire session; intervention supported parent as needed	X	X	X

O, observed; X, in session.

#### Target vocabulary word selection

Each parent worked with the SLP to select a set of target vocabulary words (words the child did not comprehend or produce in speech or manual sign), motivating and appropriate to the three activities, that would appear on the SGD or be spoken during the sessions. None of the target vocabularies included manual signs. If a child had a manual sign for a vocabulary item, it was not included as one of their target vocabulary items.

At the onset of the intervention, each child had approximately 16 target words. Examples of these target words included during play: *doll, car, push*; picture book reading/looking: *open, book, dog*; and snack: *cookie, juice, more*. Some of the target vocabulary words were specific to one of the three activities (e.g., doll) and others were used across all three activities (e.g., more). When the parent and SLP determined that the child was consistently using a target vocabulary word appropriately during the intervention sessions, new words were added either to the SGD or to the spoken vocabulary list.

### Measures

A number of measures were obtained from the children and their parents over the course of the study. First, a set of developmental assessments were administered to each child at the pre-intervention sessions. Second, intervention outcomes were measured by assessing growth in spontaneous target vocabulary production and communication use at the end of the intervention (sessions 18 and 24). Manual signs were not included in any of the measures.

#### Developmental assessments battery

The set of developmental assessments administered included measures that were directly administered to the child as well as measures that solicited parent reports of communication skills. These measures provided a profile of the children’s developmental skills. The following measures were administered: *The Mullen Scales of Early Learning* (MSEL; Mullen, 1995) is a clinician-administered developmental measure that assesses a child’s gross motor, fine motor, visual reception, and expressive and receptive language skills. The *Vineland Adaptive Behavior Scales* (VABS; Sparrow et al., 1994; VABS-II; Sparrow et al., 2005) are parent interviews that measure adaptive functioning across four domains: communication, daily living skills, socialization, and motor skills. The *Sequenced Inventory of Communication Development* (SICD; Hendrick et al., 1984) was also administered as an additional measure of expressive and receptive communication that includes direct assessment supplemented by parent reports. The *MacArthur-Bates Communicative Development Inventory: Words and Gestures* (MCDI; Fenson et al., 1993) was administered as an additional measure of parent-reported vocabulary comprehension and production, communication and language use.

#### Target vocabulary use and communication use

Language transcripts were created using the Systematic Analysis of Language Transcripts (SALT; Miller and Chapman, 1985) software program to measure the children’s target vocabulary use during the intervention at sessions 18 and 24 and communication at baseline and sessions 18 and 24. Only parent–child interactions were used for transcript creation. Transcribers were masked to the specific AC intervention but it was not possible to mask the videos from the SC group since it was clearly visible that they did not have an SDG. For a more detailed description of how the transcripts were created, see Romski et al. (2010). Reliably trained transcribers coded videos of sessions 18 and 24 for spontaneous target augmented vocabulary use, defined in the original studies as a physical indication of target vocabulary symbol use on the SGD, and target spoken vocabulary word use, defined in the original studies as a combination of sounds that were consistently and meaningfully identified by the transcriber as a target word. When describing the number and proportion of target vocabulary used, only unprompted and non-imitative spoken and augmented words were counted. Functional vocabulary is the combined total of different spoken and augmented words used. The proportion of target vocabulary was calculated by dividing the number of target vocabulary used divided by the total vocabulary available for the child’s use.

Once the transcripts were created, the SALT program provided six measures of child communication: (1) mean length of utterance in morphemes (MLU_m_ = [total number of morphemes] / [total number of utterances]), (2) mean length of turn ([total number of utterances] / [total number of turns]), (3) type/token ratio (TT ratio = [number of different words] / [total number of words used]), (4) utterance intelligibility (SALT defined utterance intelligibility as [number of intelligible words] / [number of total words]), (5) total turns (SALT defined total turns as one or more consecutive utterances), and (6) the total number of spoken and/or augmented words used as coded on the transcripts.

## Results

Table 4 reports means, standard deviations, and Mann–Whitney U results for the number and proportion of target vocabulary used in the form of spoken words, augmented symbols, and functional vocabulary. A non-parametric Mann–Whitney U test was run to determine if there were differences in children’s target vocabulary between the AC and SC intervention groups during sessions 18 and 24. Significant differences were found between children in the AC and SC groups for the number of functional vocabulary targets used, the proportion of functional vocabulary used, and the total vocabulary targets provided during intervention sessions 18 and 24. At session 18, the number of functional vocabulary targets used by children in the AC group was significantly higher (mean rank = 18.00) than the number of functional vocabulary used by children in the SC group (mean rank = 3.50), *U* = 138, *z* = 3.73, *p* = 0.000. The proportion of functional vocabulary used for children in the AC group was significantly higher (mean rank = 18.00) than the proportion of functional vocabulary used by children in the SC group (mean rank = 3.50), *U* = 138, *z* = 3.72, *p* = 0.000. The total vocabulary targets provided was significantly higher for children in the AC group (mean rank = 17.15) than children in the SC group (mean rank = 6.75), *U* = 118.50, *z* = 2.7, *p* = 0.005. At session 24, the number of functional vocabulary items used by children in the AC group continued to be significantly higher (mean rank = 18.00) than the number of functional vocabulary used by children in the SC group (mean rank = 3.50), *U* = 138, *z* = 3.72, *p* = 0.000. The proportion of functional vocabulary used for children in the AC group was significantly higher (mean rank = 18.00) than the proportion of functional vocabulary used by children in the SC group (mean rank = 3.50), *U* = 138, *z* = 3.72, *p* = 0.000. The total number of vocabulary targets available for use was significantly higher for children in the AC group (mean rank = 17.22) than children in the SC group (mean rank = 6.50), *U* = 120.00, *z* = 2.77, *p* = 0.004.

**Table 4:** Comparing target vocabulary use between children with Down syndrome in augmented communication intervention and spoken communication intervention.

	Session 18					Session 24				
Variable	AC *n* = 23	SC *n* = 6	U	z	*p*	AC *n* = 23	SC *n* = 6	U	z	*p*
No. of augmented words used	11.74 (5.43)	–				12.3 (5.47)	–			
% of target augmented words used	0.60 (0.24)	–				0.63 (0.23)	–			
% of children using spoken words	0.52	0.33	82	0.81	0.51	0.39	0.33	73	0.26	0.85
No. of different spoken words	1.70 (3.88)	0.33 (0.52)	86	1.01	0.38	1.87 (4.77)	0.67(1.21)	74	0.31	0.81
% target spoken words	0.11 (0.10)	0.06 (0.00)	13	0.18	1.00	0.15 (0.14)	0.14(0.09)	9	0.00	1.00
functional vocabulary used	12.57 (6.31)	0.33 (0.52)	138	3.75	0.00**	12.82(6.51)	0.67(1.21)	138	3.73	0.00**
% functional vocabulary used	0.63 (0.22)	0.02 (0.03)	138	3.72	0.00**	0.64 (0.23)	0.05(0.08)	138	3.72	0.00**
Total target vocabulary available	19.83 (7.51)	15.17 (0.98)	118	2.69	0.01**	19.83(7.45)	15.17(0.98)	120	2.77	0.00**

Number of augmented and spoken words and percentages are means (with standard deviations in parentheses). *p < 0.05, two-tailed; **p < 0.01, two tailed.

Table 5 reports means, standard deviations and Mann–Whitney U results for parent and child communication measures of Mean Length of Utterance in morphemes (MLUm), Mean length of turn (ML turns), total turns, type-token ratio (TTR), and child communication intelligibility. A Mann–Whitney U test was run to determine differences in child and parent communication measures in the AC SC intervention groups during baseline, session 18, and session 24. There were no significant differences between the children in the AC and SC groups at baseline for any of the measures. At session 18, children in the AC group had a significantly higher TTR, % of intelligible communication utterances, and total number of turns. Parents of children in the AC group exhibited a larger MLUm and took significantly more turns than parents of children in the SC group. By session 24, children in the AC group had a significantly longer mean length of turns than children in the SC group. Children in the AC group continued to demonstrate a significantly higher percentage of intelligible communicative utterances. Parents of children in the AC group continued to use a significantly higher MLUm to communicate with their children than parents in the SC group.

**Table 5:** Comparing communication measures between children with Down syndrome in augmented and spoken communication intervention and their parents.

	Baseline					Session 18					Session 24				
Variable	AC *n* = 23	SC *n* = 6	U	Z	*p*	AC *n* = 23	SC *n* = 6	U	z	*p*	AC *n* = 23	SC *n* = 6	U	z	*p*
Child															
MLUm	0.97 (0.05)	0.99 (0.01)	57.5	−0.63	0.55	0.99 (0.06)	0.98 (0.03)	88	1.04	0.33	1.04 (0.13)	0.92 (0.12)	100	1.68	0.10
ML turns	1.11 (0.07)	1.19 (0.16)	54.50	−0.78	0.45	1.21 (0.14)	1.13 (0.14)	101	1.73	0.09	1.20 (0.14)	1.06 (0.04)	124	2.99	0.00**
TT ratio	0.05 (0.01)	0.04 (0.03)	90	1.14	0.28	0.12 (0.06)	0.05 (0.03)	121	2.81	0.00**	0.13 (0.07)	0.11 (0.08)	82.5	0.73	0.48
intelligibility	0.07 (0.07)	0.07 (0.10)	80	0.60	0.58	0.34 (0.18)	0.06 (0.07)	134	3.51	0.00**	0.42 (0.22)	0.12 (0.10)	124.5	2.99	0.00**
total turns	124.78 (72.33)	110.17 (42.22)	71	0.11	0.94	187.17 (74.95)	101.17 (37.76)	117	2.59	0.01*	162.87 (59.32)	102.50 (73.16)	101	1.72	0.09
Parent															
MLUm	3.31 (0.41)	3.09 (0.56)	75.5	0.35	0.73	3.41 (0.33)	3.03 (0.37)	108	2.10	0.04*	3.47 (0.39)	3.18 (0.26)	108	2.10	0.04*
ML turns	17.59 (23.06)	6.19 (3.32)	106	1.99	0.05	9.12 (6.98)	6.24 (2.09)	82	0.70	0.51	9.41 (6.85)	10.84 (10.06)	74.5	0.30	0.77
total turns	118.22 (65.99)	105.50 (41.05)	68.5	−0.03	0.98	168.48 (61.30)	97.83 (32.99)	119.5	2.72	0.00**	156.00 (54.23)	105.17 (70.86)	94.00	1.35	0.19

AC, augmented communication intervention; SC, spoken communication intervention; MLUm, mean length of utterance in morphemes; TT ratio, type token ratio; Intelligibility, % of intelligible utterances; ML turns, mean length of turn in utterances; Variable means are reported (with standard deviations in parentheses). *p < 0.05, two-tailed; **p < 0.01, two tailed.

## Discussion

The children with DS who received AC intervention had stronger communication skills than the children who received the SC intervention as evidenced by significantly greater gains in functional vocabulary, and intelligible communication use. The AC interventions provided the children with a way to communicate via an SGD with visual-graphic symbols and speech output, while the children in the SC intervention were focused on producing intelligible spoken vocabulary during the activities. The AC interventions did not hinder the children’s spoken vocabulary development. Children in the AC intervention groups also maintained their gains in communication skills when the intervention transitioned from the lab environment to the home environment. Importantly, the findings from the current study suggested that the AC intervention did not hinder the spoken vocabulary development of the children with DS. Approximately half of the children with DS in the AC interventions produced spoken words by the end of intervention. In terms of utterance intelligibility, it is possible that children in the AC group may have a general advantage because words spoken with an SGD would be more intelligible than young children with only spoken word approximations.

These results are consistent with the broader findings from the two original studies that examined the parent-coached AAC interventions in a broader sample of children with developmental delays (Romski et al., 2010, 2023). These findings also support and enhance earlier case studies (e.g., Iacono and Duncum, 1995) that suggested SGDs may provide a viable communication intervention approach. It is also important to note that both the parents and children took more turns over time, which reflected a more balanced communicative exchange. It is important to note that the intervention is a combination of the use of an SGD with naturalistic communication strategies during established familiar routines. Overall, these findings suggest that SGDs are a viable intervention approach for young children with DS in the early stages of communication development. Furthermore, this study provides evidence that parents can learn to use SGDs with their children as young as 2 years of age when provided systematic coaching within naturalistic communicative exchanges.

There were some limitations to this study. First, although this study used data from two larger studies, the overall sample size of the children with DS who participated in the AC interventions was relatively small. The number of children who participated in the SC intervention was even smaller. Second, there was no comparison group of children with DS who received an AC intervention using an unaided form of AAC such as manual signs. It is not known how manual signs or PECS would have fared when compared to the AC interventions using SGDs. Additional studies are needed to carefully unpack the factors that compare the use of speech and both unaided (e.g., manual signs) and aided (e.g., SGD, PECS) forms of AAC. Third, the coding of the videos could not mask the SC vs. AC groups due to the inclusion of the SGD in the AC group interactions. It is important to note, however, that the coders were masked to the research questions and hypotheses of the studies. Fourth, parents were also taught to deliver naturalistic communication strategies as part of all the interventions. It is possible that the use of these strategies contributed to the children’s increased vocabulary and the role the inclusion of these strategies created more opportunities for the children to produce vocabulary. This can not be ruled out but all interventions included the use of naturalistic communication strategies so they could account for differences between the AC and SC groups. Finally, at baseline, standard scores on the MSEL visual reception and receptive language domains were significantly higher among children who were randomly assigned to the augmented communication condition. This may suggest more developmental delays among children who were randomly assigned to the spoken communication condition, which may also have influenced study outcomes.

In conclusion, young children with DS can benefit from a parent-implemented augmented communication intervention that incorporates technology in the form of an SGD within naturalistic communicative routines. The children with DS who received the AC interventions had stronger communication skills at the end of the 24-session intervention than the children who received the SC intervention. The AC interventions provided the children with a means to communicate via an SGD. In contrast, the children in the SC intervention were still developing their use of spoken words. At the end of the intervention, children in the AC group had a larger functional vocabulary with which to communicate and were more intelligible than the children who received the SC intervention. The AC intervention did not hinder the children’s spoken vocabulary development and in fact, was comparable, if not better than, the children’s speech development in the SC intervention.

## Data availability statement

The original contributions presented in the study are included in the article/supplementary material, further inquiries can be directed to the corresponding author.

## Ethics statement

The studies involving human participants were reviewed and approved by Insitutional Review Board, Georgia State University, Atlanta, GA. Written informed consent to participate in this study was provided by the participants’ legal guardian/next of kin.

## Author contributions

MR, RS, and AB-H contributed to the conception and design of this study and wrote additional portions of the manuscript. AB-H, CW, EF, GK, and MK organized and compiled this database from the original studies. GK performed the statistical analyses. MR, AB-H, and PA wrote the first draft of the manuscript. All authors contributed to the article and approved the submitted version.

## Funding

This research was supported in part by grant R01DC03799 from the National Institute of Deafness and Other Communication Disorders and grant R324A070122 from the United States Department of Education, Institute of Education Sciences awarded to MR. The content is solely the responsibility of the authors and does not necessarily represent the official views of the National Institutes of Health or the Institute of Education Sciences.

## Conflict of interest

The authors declare that the research was conducted in the absence of any commercial or financial relationships that could be construed as a potential conflict of interest.

## Publisher’s note

All claims expressed in this article are solely those of the authors and do not necessarily represent those of their affiliated organizations, or those of the publisher, the editors and the reviewers. Any product that may be evaluated in this article, or claim that may be made by its manufacturer, is not guaranteed or endorsed by the publisher.
